# Supplementary material for: Viral enumeration using cost-effective wet-mount epifluorescence microscopy for aquatic ecosystems and modern microbialites
Source: Appl Environ Microbiol. 2023 Nov 28;89(12):e01744-23. doi: 10.1128/aem.01744-23 (PMC10734538; doi:10.1128/aem.01744-23)
Supplement: Supplemental file 1 — Supplemental figures and tables. [file aem.01744-23-s0001.pdf]

Supplementary Data

Viral Enumeration Using Cost-Effective Wet-Mount Epifluorescence Microscopy for Aquatic Ecosystems and Modern Microbialites

Madeline Bellanger, Pieter Visscher, Richard Allen White III

Sample	Count	Normal?	SW p-value
FGL Water	19	Yes	0.05254
FGL EPS	32	Yes	0.1164
FGL Mat	25	Yes	0.9007
GSL Water	19	Yes	0.7002
GSL EPS	28	Yes	0.2201
GSL Mat	27	Yes	0.4271
FGL	76	No	0.001047
GSL	74	No	0.001769
Water	38	Yes	0.05871
EPS	60	No	0.03853
Mat	52	Yes	0.2974

Table 1. Table of samples with the amount of images counted, whether the data was normal, and the p-value from the Shapiro-Wilkes test.

Comparison	Both Normal?	Equal Variances?	F test p-value	Test Used	p-value	Significant?
FGL Water vs GSL Water	Yes	Yes	0.1585	Student's T Test	0.0002664	Yes
FGL EPS vs GSL EPS	Yes	Yes	0.8934	Student's T Test	0.5941	No
FGL Mat vs GSL Mat	Yes	Yes	0.2131	Student's T Test	0.2138	No
FGL vs GSL	No	N/A	N/A	Wilcoxon Rank Sum	0.4809	No
All Water vs All EPS	No	N/A	N/A	Wilcoxon Rank Sum	2.20E-16	Yes
All Water vs All Mat	Yes	No	2.20E-16	Welch's T test	2.20E-16	Yes
All EPS vs All Mat	No	N/A	N/A	Wilcoxon Rank Sum	1.56E-07	Yes
FGL Water vs FGL EPS	Yes	No	2.87E-08	Welch's T test	1.05E-15	Yes
FGL Water vs FGL Mat	Yes	No	1.33E-15	Welch's T test	2.20E-16	Yes
FGL EPS vs FGL Mat	Yes	No	1.09E-05	Welch's T test	5.09E-06	Yes
GSL Water vs GSL EPS	Yes	No	8.93E-13	Welch's T test	1.02E-13	Yes
GSL Water vs GSL Mat	Yes	No	2.20E-16	Welch's T test	5.75E-16	Yes
GSL EPS vs GSL Mat	Yes	No	0.0005128	Welch's T test	4.50E-05	Yes

Table 2. Table of all comparisons made, whether both datasets were normal, whether the variances were equal, the p-value of the F test, the statistical test used in the comparison, the p-value from that test, and whether the test revealed a statistically significant difference in the two datasets.

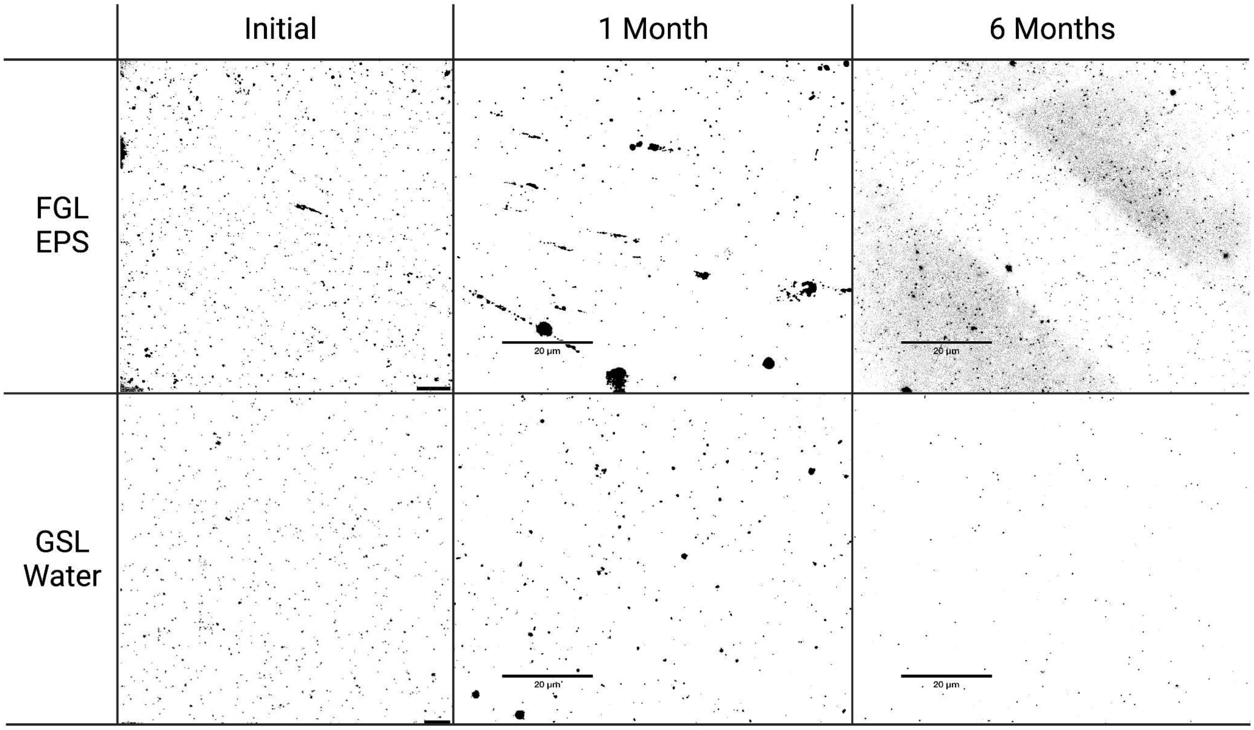

Figure 1. Time comparison of sample stability once placed on a slide. Initial images were taken on an Olympus IX83 microscope, while 1 Month and 6 Month images were taken on an ECHO Revolve. Field of view and scale bars are different on these two microscopes. Taking images in the exact same spot months apart is not easily accomplished, however, images were taken on the same slide and within the same field for each time period. Images were converted to negatives to increase visibility of VLPs.

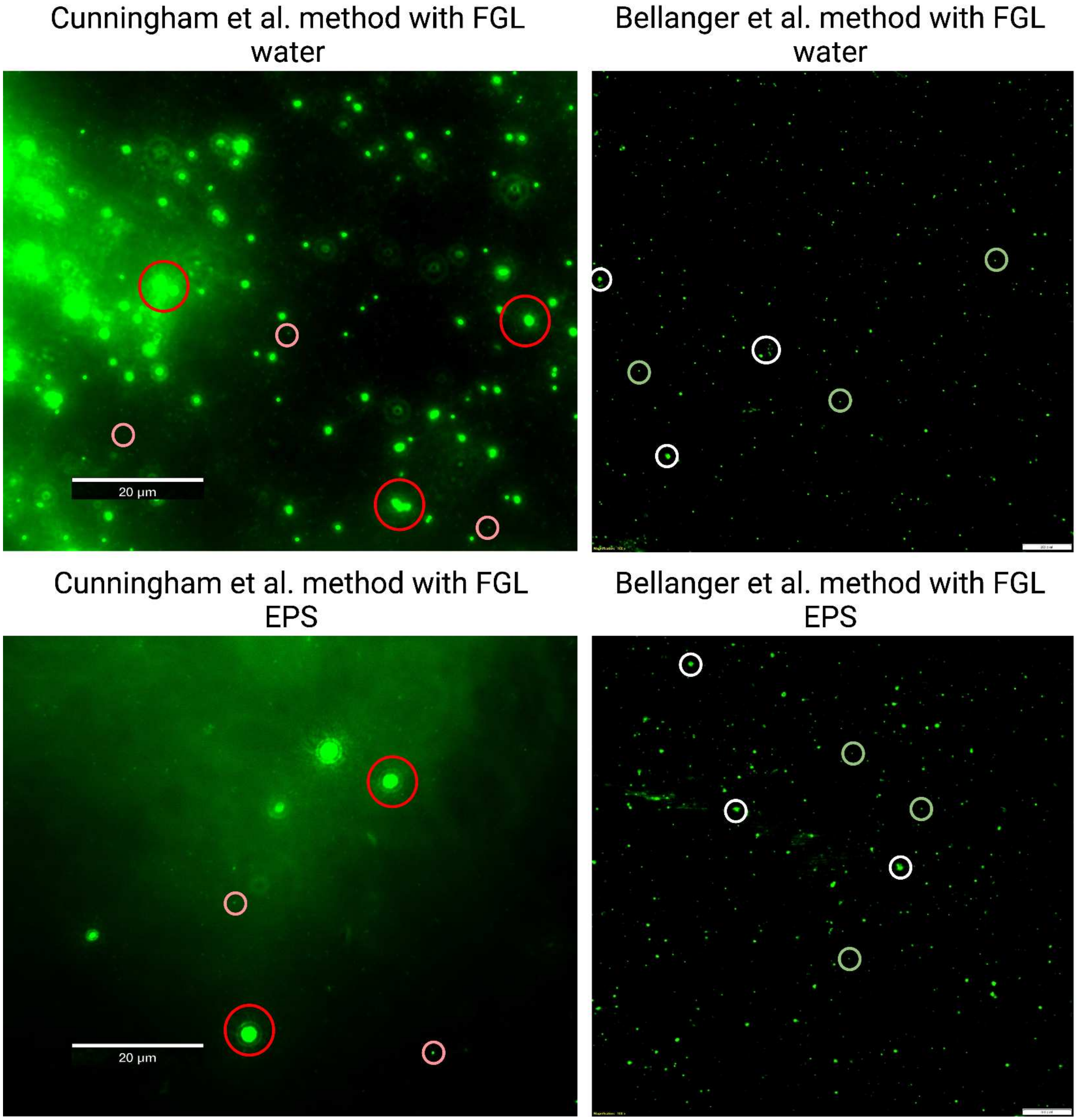

Figure 2. Methodology comparison between the Cunningham et al. method and the Bellanger et al. method. Darker red circles in Cunningham et al. images are large portions of cellular debris. Lighter red circles in Cunningham et al. images are virus-like particles. White circles in Bellanger et al. images are small portions of cellular debris. Light green circles in Bellanger et al. images are virus-like particles.

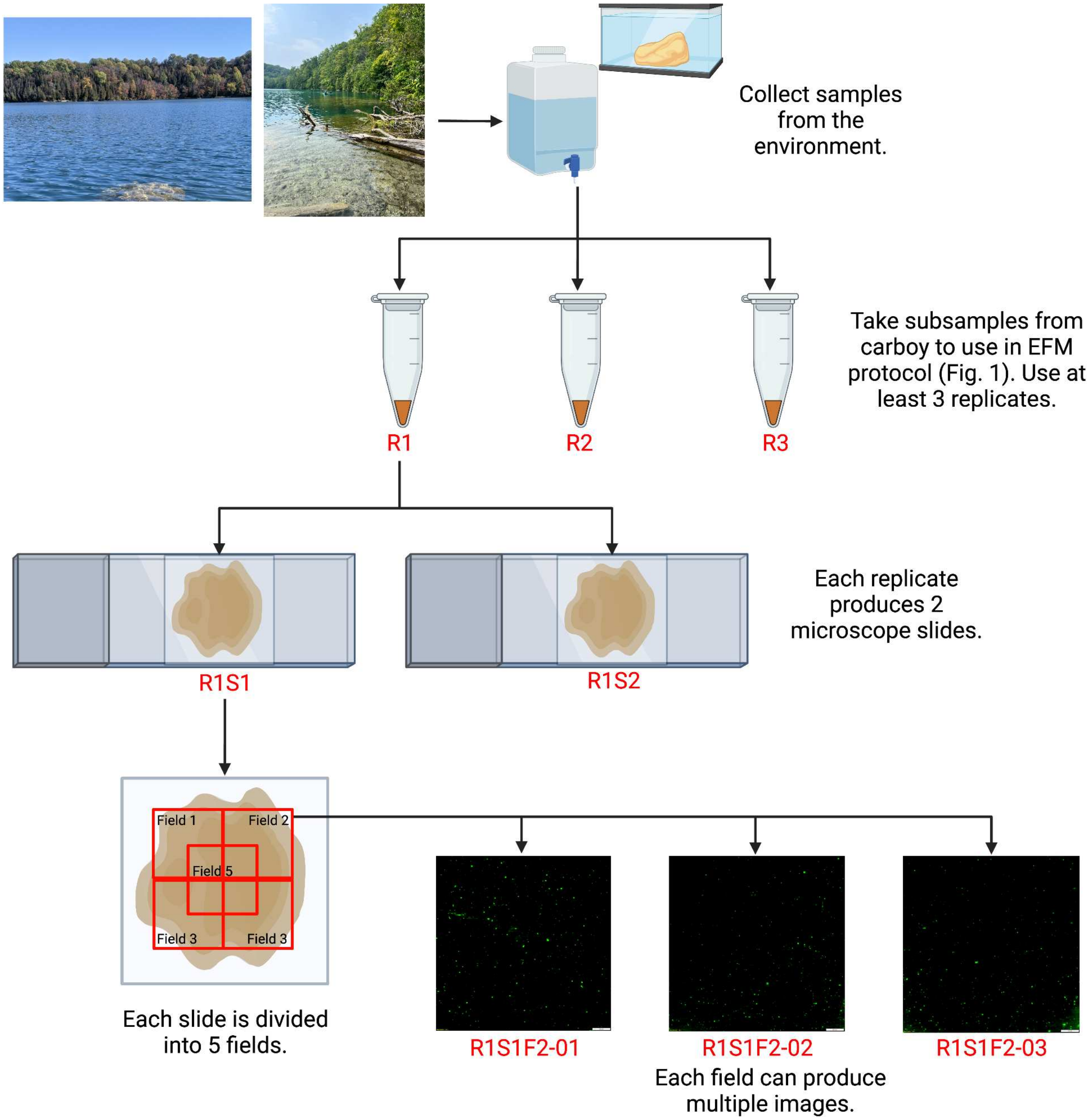

Figure 3. Breakdown of samples vs replicates vs fields vs images. Large portions (~8 inches in width) of microbial mats are taken from the environment, along with ~3 L of water. Subsamples can then be collected as needed in the lab without having to return to the sampling site. Each subsample used within experiments is referred to as a "replicate" (R#). There are multiple replicates for each sample type. Each replicate will produce two microscope slides (R#S#). During imaging, the sample on the microscope slide is divided into 5 fields (R#S#F#). Each field can have multiple images taken within it (R#S#F#_#). Fields can also overlap. Multiple images from each field are taken to ensure a proper representation of VLPs in the sample can be observed.

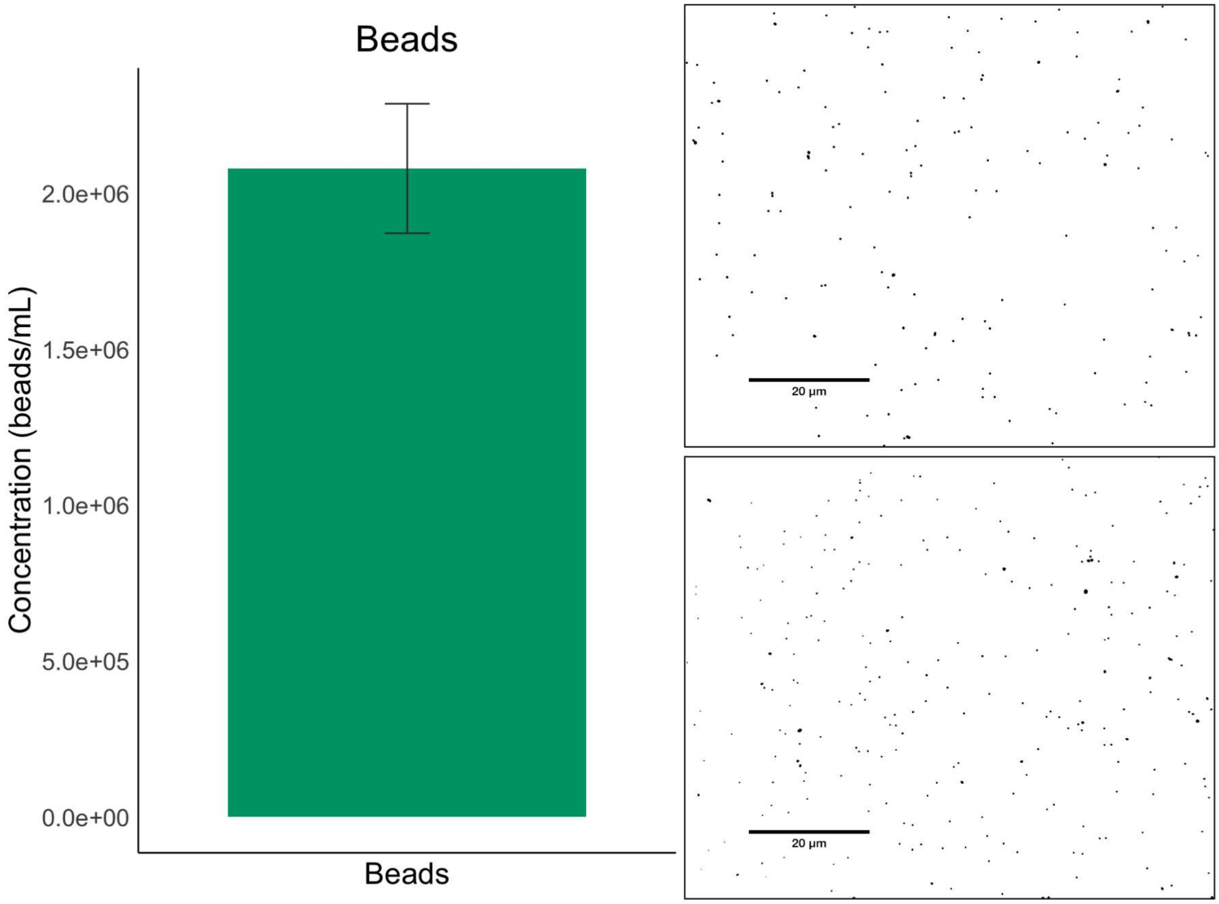

Figure 4. Enumeration of beads. Beads are 175 ± 5 nm DAPI fluorospheres. Beads are roughly the same size as virus-like particles. Images are under a 100x oil immersion with a scale bar equal to 400 pixels or 20 µm.

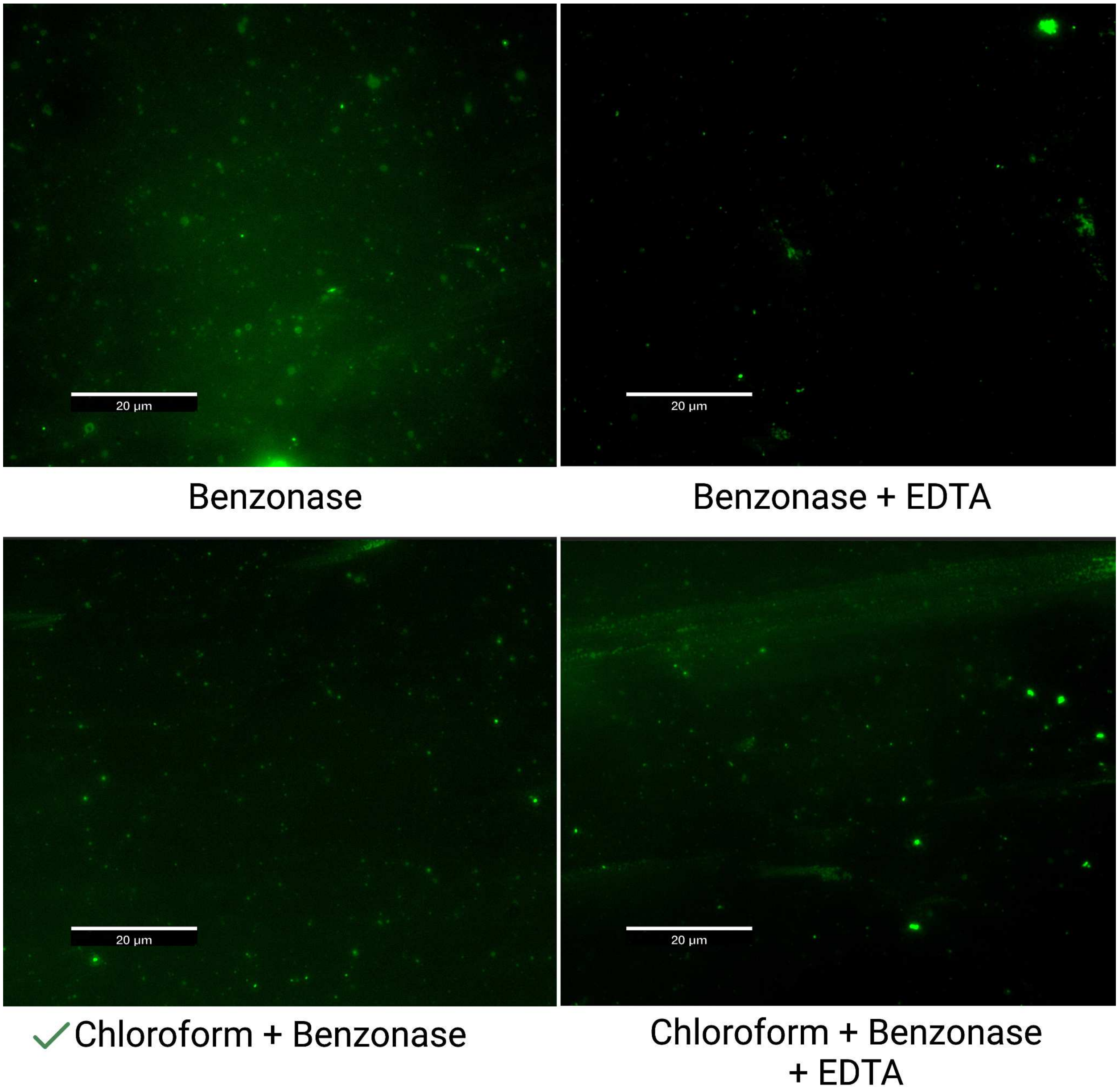

Figure 5. Comparison of water sample methodologies. Different chemicals were tested to optimize the protocol. The best combination of chemicals was chloroform and benzonase (shown with a check mark). Other methods were rejected due to large particles being present, lots of haze being observed, or minimal viral particles being observed.

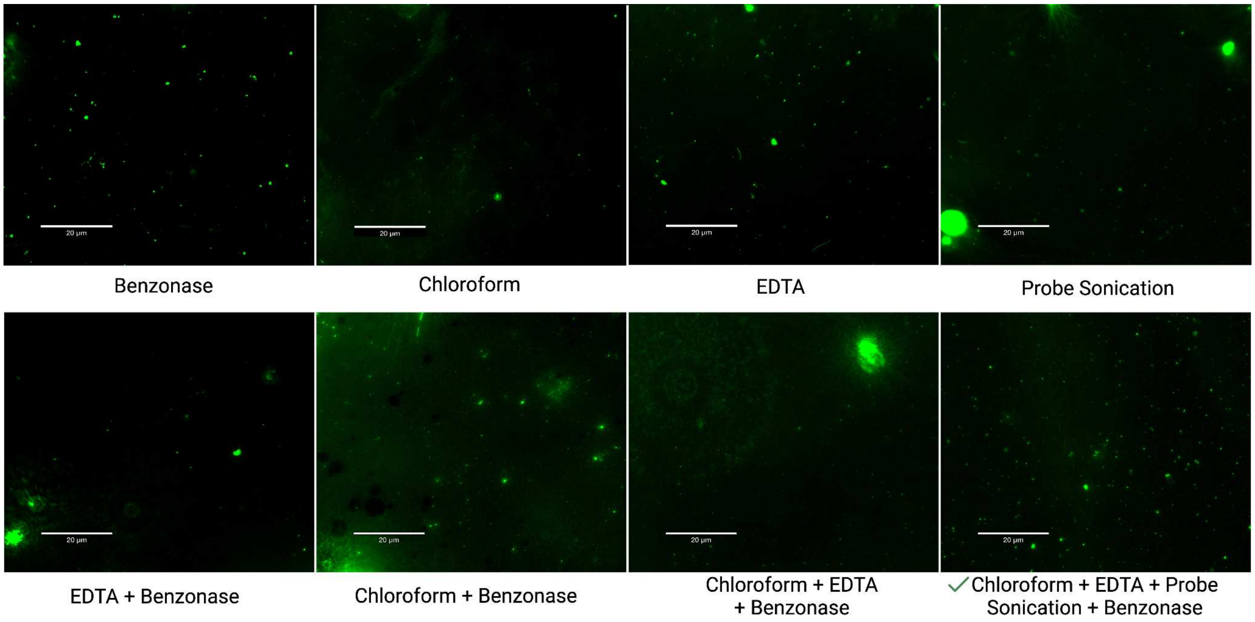

Figure 6. Comparison of EPS sample methodologies. Different chemicals and agitation methods were tested to optimize the protocol. The best combination of chemicals and agitation was chloroform, probe sonication, EDTA, and benzonase (shown with a check mark). Other methods were rejected due to large particles being present, lots of haze being observed, or minimal viral particles being observed.

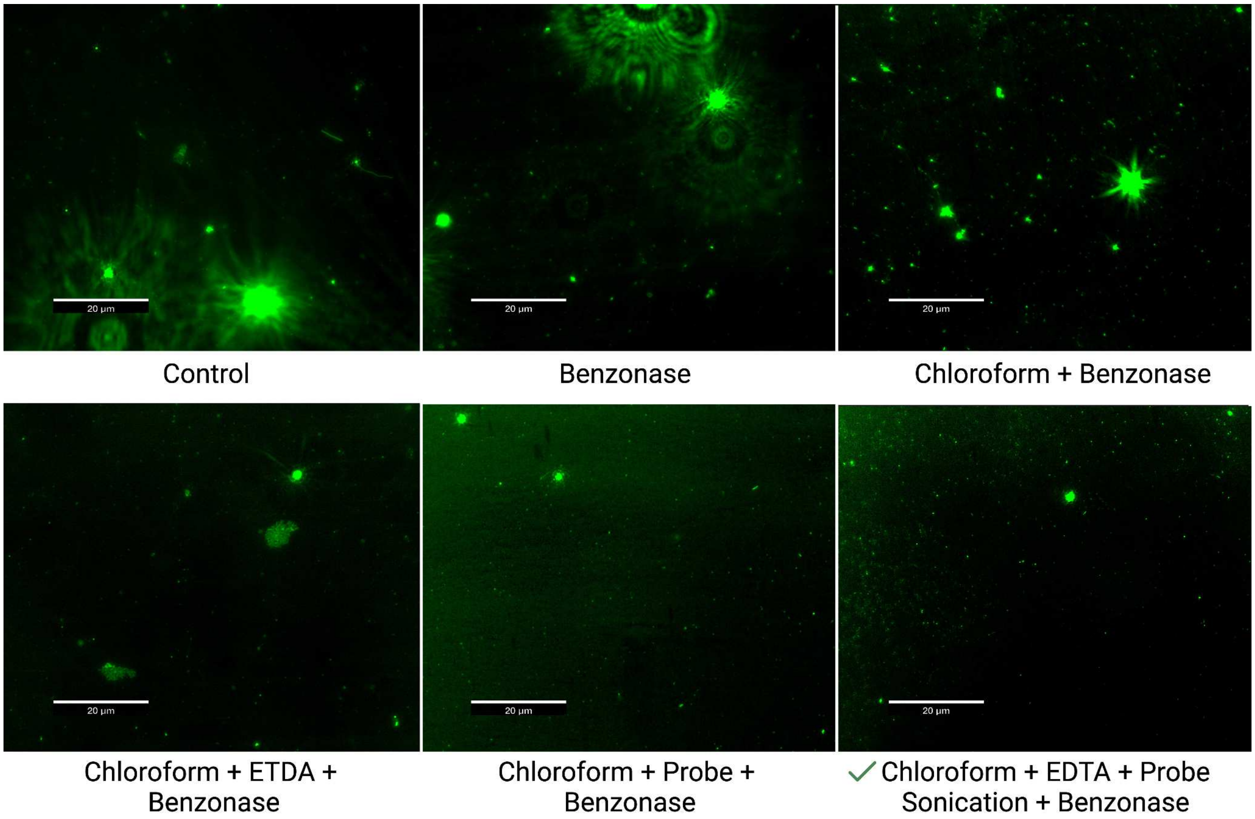

Figure 7. Comparison of mat sample methodologies. Different chemicals and agitation methods were tested to optimize the protocol. The best combination of chemicals and agitation was chloroform, probe sonication, EDTA, and benzonase (shown with a check mark). Other methods were rejected due to large particles being present, lots of haze being observed, or minimal viral particles being observed.
